# Uncontrollable uterine atony after replacement of uterine inversion managed by hysterectomy: a case report

**DOI:** 10.1186/s13256-020-02528-0

**Published:** 2020-10-08

**Authors:** Daisuke Katsura, Suzuko Moritani, Shunichiro Tsuji, Kounosuke Suzuki, Kazutaka Yamada, Mizuki Ohashi, Fuminori Kimura, Takashi Murakami

**Affiliations:** 1 Department of Obstetrics and Gynecology, Shiga University of Medical Science Hospital, Setatsukinowa-cho, Otsu, Shiga 520-2192 Japan; 2 Department of Diagnostic Pathology, Shiga University of Medical Science Hospital, Setatsukinowa-cho, Otsu, Shiga 520-2192 Japan

**Keywords:** uterine inversion, replacement, atony of uterus, reperfused blood flow, ischemic uterus, case report

## Abstract

**Background:**

Uterine inversion may cause massive hemorrhage, resulting in maternal deterioration and death. Replacement of the inverted uterus must be performed as soon as possible. As time passes, the inverted uterus becomes atonic and necrotic, and a surgical approach may be required.

**Case presentation:**

A 27-year-old Japanese woman was admitted to our hospital 4 hours postpartum with increased hemorrhage after the replacement of an inverted uterus. Recurrent inversion was diagnosed, and though the atonic uterus was replaced again by the Johnson maneuver, hemorrhage persisted. Balloon tamponade was not successful in stopping the hemorrhage, and uterine artery embolization was performed. Bleeding resumed the next day on removal of the balloon, and hysterectomy was performed. Massive hemorrhage, coagulopathy, and uterine necrosis caused uterine atony, and the reperfused blood flow on replacement of the ischemic uterus increased hemorrhage.

**Conclusions:**

Cases of uterine inversion with coagulopathy lasting for more than 4 hours may require a surgical intervention, and uterine replacement may have to be delayed until the maternal hemodynamic condition is stabilized. Uterine replacement under laparotomy may be also be considered due to the risk of increased hemorrhage.

## Background

Although uterine inversion is a rare obstetric emergency and occurs in only 1 in 20,000 vaginal births, it causes acute maternal blood loss and can lead to maternal death [1]. Therefore, prompt management with fluid resuscitation and control of hemorrhage is important. In particular, the inverted uterus must be replaced as soon as possible because the success rate decreases with the involution of the cervix, which induces a rigid ring that makes restoration of the normal uterus position difficult [2, 3]. As time passes, the uterus becomes atonic and necrotic, and a surgical approach may be required [2–4]. However, the time from inversion to the occurrence of uterine atony and necrosis is uncertain.

We encountered a case of a patient with increased hemorrhage and coagulopathy after the replacement of a uterine inversion that had persisted for 4 hours.

## Case presentation

A 27-year-old Japanese woman with unremarkable medical and family history was impregnated through *in vitro* fertilization. At 40 weeks of gestation, she was admitted to an obstetric clinic for premature rupture of the membranes and vaginal delivery by induced labor. After delivery, a large mass emerged through the vaginal passage with the placenta, and the placenta was removed. Uterine inversion was diagnosed, and manual repositioning of the uterus was performed with oxytocin administration. However, because hemorrhage persisted, she was referred to our hospital 4 hours postpartum.

**Fig. 1:**
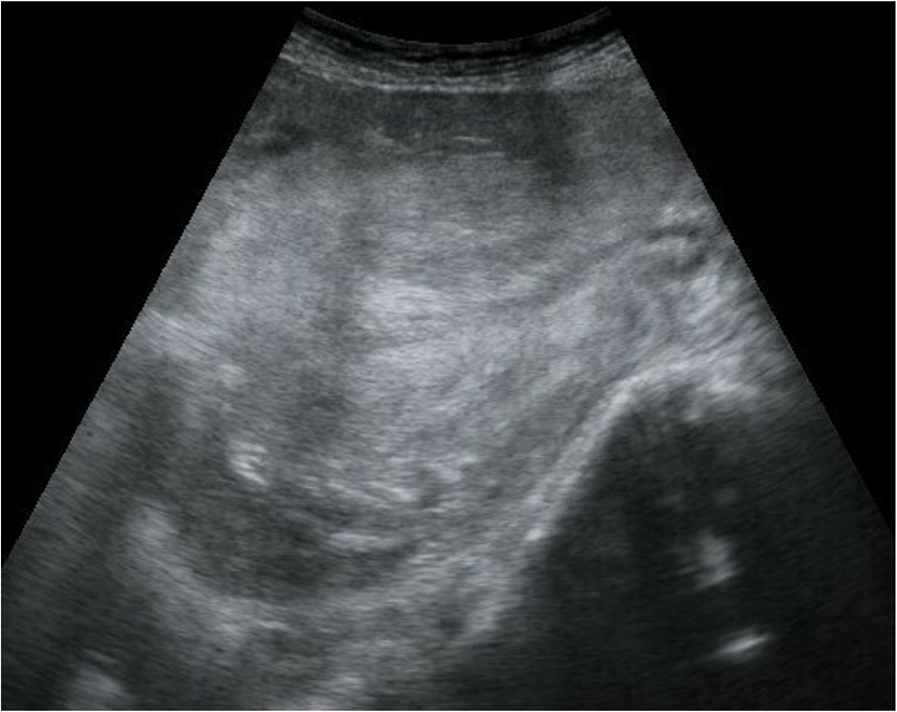
Abdominal ultrasonography findings. The uterine fundus passes through the cervical ring (sagittal view: right side – patient’s head, left side – patient’s leg)

On admission, she had lost over 4000 mL of blood, was unconscious, and her blood pressure could not be measured. Laboratory tests revealed hemoglobin, platelet, and fibrinogen levels of 3.4 g/dl, 18.2 × 10^4^/μl, and 131 mg/dl, respectively. We started rapid blood transfusion, intubated her, administered oxygen, and diagnosed uterine inversion by vaginal examination and abdominal ultrasonography (Fig. 1). Although we successfully replaced the inverted uterus using the Johnson maneuver followed by internal bimanual compression, the uterus was markedly atonic and the hemorrhage increased. Balloon tamponade was performed to stop the hemorrhage and reduce the risk of recurrence. However, hemorrhage persisted at a rate of 350 mL/min. She went into cardiopulmonary arrest and was resuscitated after 11 minutes. She presented with a pulse rate of 140 beats per minute (bpm) and a blood pressure of 70/40 mmHg. Laboratory tests revealed hemoglobin, platelet, and fibrinogen levels of 5.3 g/dl, 3.9 × 10^4^/μl, and 116 mg/dl, respectively. To stop the hemorrhage immediately, uterine artery embolization was performed instead of hysterectomy because of disseminated intravascular coagulation, and the hemorrhage stopped. She presented with a pulse rate of 100 bpm and a blood pressure of 120/70 mmHg. Laboratory tests revealed hemoglobin, platelet, and fibrinogen levels of 9.5 g/dl, 6.8 × 10^4^ /μl, and 147 mg/dl, respectively. She required transfusion of 38 units of fresh frozen plasma and 22 units of packed red blood cell concentrate. When the balloon was removed one day later, bleeding increased again, and she experienced a hemorrhage of 500 ml in 2 hours. Therefore, a hysterectomy was performed, and her condition stabilized. She presented with a pulse rate of 80 bpm and a blood pressure of 140/70 mmHg. Laboratory tests revealed hemoglobin, platelet, and fibrinogen levels of 8.6 g/dl, 8.6 × 10^4^ /μl, and 297 mg/dl, respectively. Subsequently, computed tomography revealed brain edema and a fogging effect in the cortico-medullary junction, and she was diagnosed with postresuscitation encephalopathy. Her cerebral activity potential could not be confirmed, and communication with her is difficult, although she opens her eyes spontaneously and responds slightly to painful stimulation.

Placental and uterine histopathological examination revealed placenta accreta on part of the uterine fundus (because of no decidua) and necrosis in the fundus and on the surface of the surrounding muscle layer.

## Discussion 

When uterine inversion occurs, we must replace the inverted uterus immediately before a uterine contraction ring forms [2, 3]. Close follow-up is required because uterine inversion can recur [4, 5]. In addition, surgery may be required if the uterus becomes atonic and necrotic [2–4]. In our case, the persistent hemorrhage suggested recurrent uterine inversion after manual repositioning of the uterus. Although there was a 4-hour delay from the recurrent uterine inversion, we easily replaced the uterus. However, the uterus did not contract at all, and hemorrhage increased. We speculated that the replacement was easy because massive hemorrhage and concomitant coagulopathy might have caused remarkable atony of the uterus [6, 7]. However, hemorrhage increased on replacement of the uterus because the blood flow was reperfused into the ischemic uterus. Therefore, in cases of uterine inversion with coagulopathy that persists for a significant period, uterine replacement may have to be delayed until after fluid resuscitation and blood transfusion can stabilize the maternal hemodynamic condition. Furthermore, uterine replacement under laparotomy may be considered because hemorrhage may increase after uterine replacement; thus, we can promptly perform a uterine compression suture, stepwise uterine devascularization, or/and hysterectomy.

In addition, uterine necrosis may result from uterine atony. However, the time from uterine inversion to the development of necrosis is unknown. A previous study in the uteri of cynomolgus macaques showed that ischemia for 4 hours caused mild muscle degeneration and zonal degeneration. Periodical menstruation resumed in all animals with ischemia up to 4 hours, but animals with ischemia for 8 hours did not recover menstruation and had atrophic uteri [8]. In our case, uterine inversion persisted for up to 4 hours, and although it is necessary to consider the influence of balloon tamponade and uterine artery embolization, necrosis was observed in the uterine fundus and on the surface of the surrounding muscle layer. Four hours might be the critical time from the occurrence of uterine inversion to the probable development of necrosis, but this requires further study.

In our case, we first performed balloon tamponade and uterine artery embolization instead of hysterectomy due to the risk of perioperative hemorrhaging secondary to disseminated intravascular coagulation and the momentum of bleeding. Had we stabilized her hemodynamic condition before replacement of the inverted uterus or replaced the inverted uterus under laparotomy, we may have been able to perform hysterectomy promptly even if the bleeding increased.

## Conclusions

In summary, acute uterine inversion generally needs prompt replacement of the inverted uterus. However, when uterine inversion with coagulopathy persists for more than 4 hours, clinicians should consider stabilizing the maternal hemodynamic condition before replacement of the inverted uterus or should consider a surgical approach without undue delay. To the best of our knowledge, this is the first case report on a novel management strategy of acute uterine inversion with coagulopathy that persisted for a significant period of time.

## Data Availability

Not applicable.
